# Effects of Metformin on the Cerebral Metabolic Changes in Type 2 Diabetic Patients

**DOI:** 10.1155/2014/694326

**Published:** 2014-03-24

**Authors:** Yung-Cheng Huang, Chien-Chin Hsu, Wei-Che Lin, Tang-Kai Yin, Chi-Wei Huang, Pei-Wen Wang, Han-Hsuan Chang, Nan-Tsing Chiu

**Affiliations:** ^1^Department of Nuclear Medicine, Kaohsiung Chang Gung Memorial Hospital and Chang Gung University College of Medicine, 123 Dapi Road, Niaosong District, Kaohsiung 833, Taiwan; ^2^Department of Diagnostic Radiology, Kaohsiung Chang Gung Memorial Hospital and Chang Gung University College of Medicine, 123 Dapi Road, Niaosong District, Kaohsiung 833, Taiwan; ^3^Department of Computer Science and Information Engineering, 700 Kaohsiung University Road, Nanzih District, Kaohsiung 811, Taiwan; ^4^Department of Neurology, Kaohsiung Chang Gung Memorial Hospital and Chang Gung University College of Medicine, 123 Dapi Road, Niaosong District, Kaohsiung 833, Taiwan; ^5^Division of Endocrinology and Metabolism, Department of Internal Medicine, Kaohsiung Chang Gung Memorial Hospital and Chang Gung University College of Medicine, 123 Dapi Road, Niaosong District, Kaohsiung 833, Taiwan; ^6^Department of Nuclear Medicine, National Cheng Kung University Hospital, College of Medicine, National Cheng Kung University, 138 Shengli Road, Tainan 70428, Taiwan

## Abstract

Metformin, a widely used antidiabetic drug, has numerous effects on human metabolism. Based on emerging cellular, animal, and epidemiological studies, we hypothesized that metformin leads to cerebral metabolic changes in diabetic patients. To explore metabolism-influenced foci of brain, we used 2-deoxy-2-[^18^F]fluoro-D-glucose (FDG) positron emission tomography for type 2 diabetic patients taking metformin (MET, *n* = 18), withdrawing from metformin (wdMET, *n* = 13), and not taking metformin (noMET, *n* = 9). Compared with the noMET group, statistical parametric mapping showed that the MET group had clusters with significantly higher metabolism in right temporal, right frontal, and left occipital lobe white matter and lower metabolism in the left parahippocampal gyrus, left fusiform gyrus, and ventromedial prefrontal cortex. In volume of interest (VOI-) based group comparisons, the normalized FDG uptake values of both hypermetabolic and hypometabolic clusters were significantly different between groups. The VOI-based correlation analysis across the MET and wdMET groups showed a significant negative correlation between normalized FDG uptake values of hypermetabolic clusters and metformin withdrawal durations and a positive but nonsignificant correlation in the turn of hypometabolic clusters. Conclusively, metformin affects cerebral metabolism in some white matter and semantic memory related sites in patients with type 2 diabetes.

## 1. Introduction

Metformin, (N,N-dimethylimidodicarbonimidic diamide hydrochloride; commercial name: Glucophage, Glumetza, Riomet), a widely used antihyperglycemic drug administered orally for the management of type 2 diabetes, has numerous effects on human metabolism [1, 2]. It has the ability to decrease hepatic glucose production and intestinal absorption and improve insulin sensitivity by increasing peripheral glucose uptake and utilization. Additionally, it lowers blood cholesterol and triglyceride levels, reducing the risk of developing heart disease. Unlike sulfonylureas, metformin does not cause hyperinsulinemia in either type 2 diabetic patients or normal subjects, insulin secretion remaining unchanged. The association between metformin and dementia has recently been reported in cellular models, animal models, and epidemiological studies. Metformin can cross the blood-brain barrier and have specific pharmacological effects on the central nervous system (CNS) [3]. The exact mechanism and sites of its action in the CNS remain uncertain, however.

Metformin has recently attracted much attention because of its possibly beneficial effects on the CNS. Metformin may attenuate CNS-based inflammation, protect against apoptotic cell death in primary cortical neurons, and promote neurogenesis, and it is a potential therapy for injured or degenerating nervous system in cellular and animal models [4–7]. In contrast, one study [8] reported that metformin could deregulate**β**-secretase (BACE1) promoter activity and induce more than twice the normal production of**β**-amyloid peptide (A**β**), the protein that forms toxic brain plaques in Alzheimer's disease (AD).

Epidemiological evidence [9, 10] suggests that diabetes increases the risk of dementia; diabetes and dementia are two of the most common and devastating health problems in the elderly. “Diabetes dementia” is probably a mix of vascular and neurodegenerative dementia. Two population-based epidemiological studies about dementia and metformin administration had contrasting results: a decreased risk of dementia observed in Taiwan [11], but a slightly increased risk in long-term users in the UK [12].

Based on these studies, we hypothesized that metformin causes cerebral metabolic changes in type 2 diabetic patients. Because a lack of human data about metformin's effects on cerebral metabolism, we used 2-deoxy-2-[^18^F]fluoro-D-glucose (FDG) positron emission tomography (PET) to scan patients with type 2 diabetes and then analyzed the scans using statistical parametric mapping (SPM).

## 2. Materials and Methods

This study was approved by the Institutional Review Board of Chang Gung Memorial Hospital.

### 2.1. Patients

Adults with type 2 diabetes who were taking antidiabetic drugs were recruited prospectively. Exclusion criteria were neuropsychological or medical conditions that can alter mental status, alcohol or substance abuse, using hypnotics during the previous 2 weeks, brain tumor, autoimmune disease, AIDS, pregnancy, and a history of head trauma with a loss of consciousness. Detailed information about the study was given to all participants, and all signed written informed consent forms before they entered the study. At the visits, medical and trauma histories were reviewed, glycated hemoglobin (HbA_1c_) levels were checked, and the Mini-Mental State Examination (MMSE) was administered and the scores recorded. Finally, 40 patients were enrolled. Based on their metformin usage, they were categorized into three groups: (i) MET: patients taking metformin (*n* = 18); (ii) wdMET: patients who were taking metformin but had begun withdrawing from it more than 3 days before the FDG PET scan (*n* = 13); and (iii) noMET: patients who had not taken metformin (*n* = 9). All participants, including the patients not taking metformin (noMET), were subjected to FDG PET scan for the purpose of cancer screening. This study is not considered a clinical drug trial but a clinical observational study.

### 2.2. FDG PET

FDG PET studies were done using a combined PET/CT scanner (Discovery ST; GE Healthcare, Waukesha, WI, USA). The blood glucose cutoff level that contraindicated FDG injection was 11.1 mmol/L. Supplemental insulin was not given to lower the blood sugar just before the FDG injection because we were concerned that the insulin would alter the FDG distribution. Participants fasted for at least 6 h before they were injected with 370–555 MBq (10–15 mCi) of FDG. They then rested for 60 min in a quiet room, with the lights dimmed and their eyes closed. The participants were instructed to refrain from reading, listening to music, and talking during the uptake period. The brain scan consisted of a 1-field-of-view CT scan followed by a 15-min PET study in 2D mode. Transaxial 2D PET data from the brain scan were reconstructed using an ordered subsets expectation maximization algorithm (OSEM) (2 iterations, 30 subsets) as 128 × 128-pixel images and a slice thickness of 3.27 mm. The CT data were used for PET attenuation correction.

### 2.3. SPM Analyses

SPM5 (Wellcome Department of Imaging Neuroscience, University College of London, UK) implanted in MATLAB 7.7 (The MathWorks, Natick, MA, USA) was used to analyze images. PET images were interpolated (trilinearly) to a size of 2 × 2 × 2 mm voxels, spatially normalized to the standard PET template embedded in SPM5 and then smoothed with a Gaussian kernel (full-width at half-maximum = 10 mm). Global normalization and proportional scaling with 0.8 threshold masking were applied. The three-way voxel-wise analyses were compared using two-sample *t*-tests in a covariance model that included age, body mass index (BMI), fasting blood sugar, and years of education as nuisance variables. *T* contrast was used, and the results were at threshold with *P* < 0.005 (uncorrected for multiple comparisons), a criterion used in several previous studies [13, 14], with an extent threshold of 150 voxels (1 voxel = 8 mm^3^) over whole brain regions. The results were displayed using the SPM5 extension xjView 8.1 (http://www.alivelearn.net/xjview8/), the peak coordinates for each significant cluster were labeled for specific anatomic location using Talairach Daemon 2.4.2 (http://www.talairach.org/), and tissue type (both white and gray matter) was verified by visual inspection. We used the SPM5 extension MarsBaR toolbox 0.43 (http://marsbar.sourceforge.net/) to extract individual adjusted, normalized FDG uptake values from the eligible clusters showing significant differences in the voxel-wise comparison between the MET and noMET groups. The regional FDG uptake values were then used to evaluate the differences between the three groups and to analyze correlations with the time intervals from the last dose of metformin to the FDG injection (withdrawal durations) in the MET and wdMET groups.

### 2.4. Statistical Analyses

Differences in gender, age, BMI, HbA_1c_, fasting blood sugar, metformin daily dose, diabetes duration, MMSE score, and education between groups were compared using a *χ* ^2^ test and one-way analysis of variance (ANOVA), as appropriate. Group differences in FDG uptake values in eligible clusters were examined using one-way ANOVA followed by two-tailed post hoc Student's *t*-test. Associations between normalized FDG uptake values in eligible clusters and metformin withdrawal durations were assessed using Pearson correlations. SPSS 20 for Windows (SPSS Inc., Chicago, IL, USA) was used for the statistical analyses. Significance was set at *P* < 0.05.

## 3. Results

### 3.1. Patient Characteristics

All patients were right-handed. There were no significant differences in gender, age, BMI, HbA_1c_, fasting blood sugar, diabetes duration, MMSE score, education, or smoking between groups (Table 1). The prescribed daily dose of metformin was not significantly different between the MET and wdMET groups.

### 3.2. Voxel-Wise SPM between Group Comparisons

**Figure 1:**
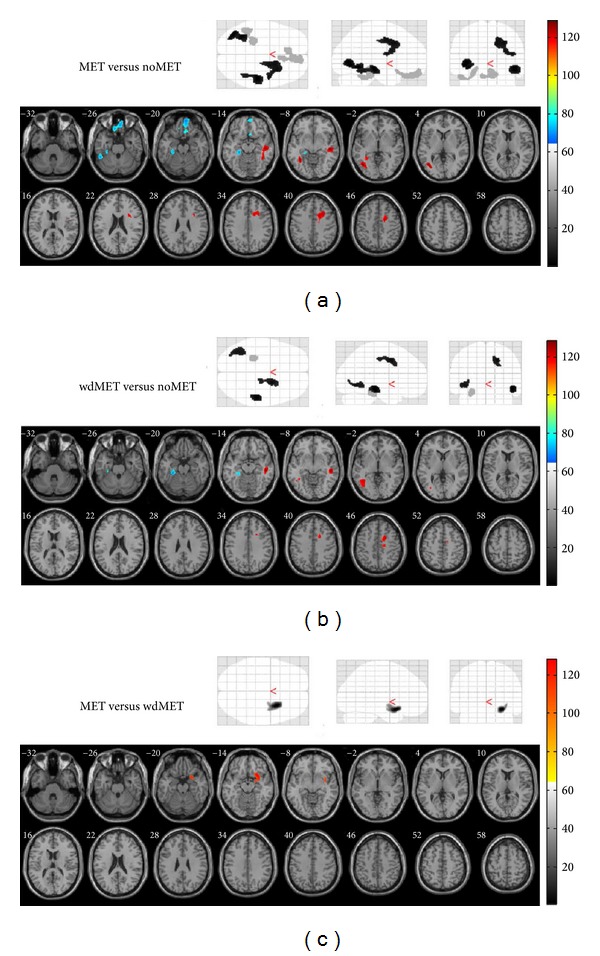
SPM analysis to compare cerebral FDG uptake between MET-group patients (taking metformin), noMET-group patients (not taking metformin), and wdMET-group patients (withdrawing from metformin). (a) MET versus noMET; (b) wdMET versus noMET; and (c) MET versus wdMET. The voxel-wise comparisons included age, body mass index, fasting blood sugar, and education as nuisance variables. Top: SPM glass brain render of the statistical voxel-wise comparisons (black: hypermetabolic regions in the former; light gray: hypometabolic regions in the former). Bottom: SPM results, slice renders of the significant clusters displayed on a T1 template overlaid with magnetic resonance images by SPM5 extension xjView 8.1 (red: hypermetabolic regions in the former; blue: hypometabolic regions in the former). All results are presented at a threshold of *P* < 0.005, uncorrected, *k* > 150 voxels.

The MET group had areas with significantly (*P* < 0.005, uncorrected, *k* > 150) higher metabolism than did the noMET group in the white matter of the right temporal, right frontal, and left occipital lobes (Figure 1(a)); the wdMET group had areas with significantly (*P* < 0.005, uncorrected, *k* > 150) higher metabolism in the same areas than did the noMET group, but they were smaller than those in the MET group (Figure 1(b)). In the MET group, metabolism was significantly (*P* < 0.005, uncorrected, *k* > 150) lower in the parahippocampal gyrus (PH) of the left limbic lobe, the fusiform gyrus (FG) of the left temporal lobe (Brodmann area 37), and the ventromedial prefrontal cortex (VMPFC), including foci in the right orbital gyrus (Brodmann area 11), left rectal gyrus (Brodmann area 11), and right medial frontal gyrus (Figure 1(a)) than in the noMET group. In the wdMET group, metabolism was significantly (*P* < 0.005, uncorrected, *k* > 150) lower than in the noMET group only in the PH of the left limbic lobe (Figure 1(b)). In the comparison between the MET and wdMET groups, SPM analysis detected subtle differences in only one white-matter focus. Compared with the wdMET group, the MET group showed an area with significantly (*P* < 0.005, uncorrected, *k* > 150) higher metabolism in the white matter of the right frontal lobe (Figure 1(c)); however, there was no suprathreshold cluster of lower metabolism.

### 3.3. Volume of Interest (VOI-) Based Group Comparisons and Correlation Analyses

**Figure 2:**
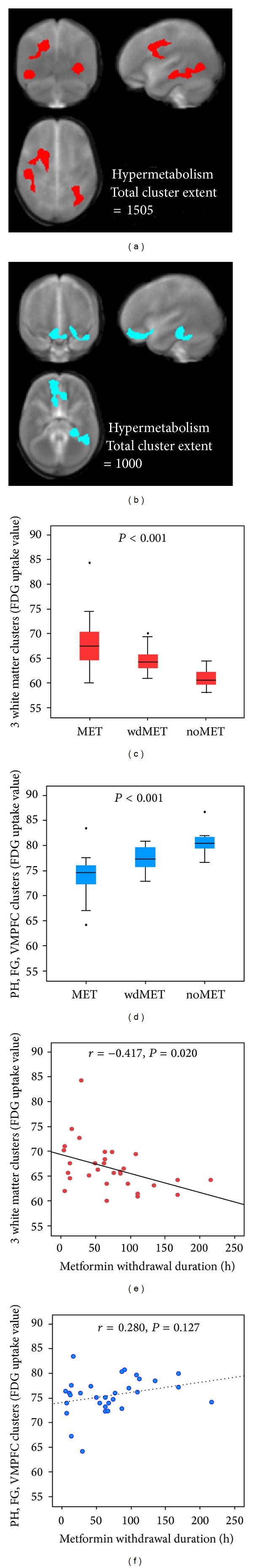
VOI-based (Top) group comparisons (middle) and correlation analyses (bottom).* Top*: (a) the red VOIs overlaid on volume rendering magnetic resonance images represent hypermetabolic clusters obtained from the voxel-wise comparison (MET > noMET, *P* < 0.005, uncorrected, *k* > 150 voxels). (b) The blue VOIs overlaid on volume rendering magnetic resonance images represent hypometabolic clusters obtained from the voxel-wise comparison (MET < noMET, *P* < 0.005, uncorrected, *k* > 150 voxels). Middle: VOI-based group comparisons between the MET (*n* = 18), wdMET (*n* = 13), and noMET (*n* = 9) groups. Box plots (median, interquartile range, and extreme values) for normalized FDG uptake values in hypermetabolic clusters (c) and in hypometabolic clusters (d) between the 3 groups (*P* < 0.001). Bottom:VOI-based correlation analyses across the MET and wdMET groups. The normalized FDG uptake values in the hypermetabolic clusters (e) and in the hypometabolic clusters (f) of the patients plotted against their metformin withdrawal durations. The solid line is a significant linear regression line (*r* = − 0.417, *P* = 0.020) and the dotted line is an estimated linear regression line (*r* = 0.280, *P* = 0.127). FG: fusiform gyrus; MET: patients taking metformin; noMET: patients not taking metformin; PH: parahippocampal gyrus; VMPFC: ventromedial prefrontal cortex; VOI: volume of interest; wdMET: patients withdrawing from metformin for more than 3 days.

Based on the results of the SPM group comparison between the MET and noMET groups (Table 2), we used the statistically significant clusters as VOIs (Figures 2(a) and 2(b)) and extracted the FDG uptake values in those eligible clusters from the individual normalized images using the MarsBaR toolbox. There were significant differences (*P* < 0.001) in the average FDG uptake values of white-matter clusters between the three groups. From the MET group to the wdMET group to the noMET group, there was a clear tendency toward lower FDG uptake values (Figure 2(c)). Within the VOIs, the average FDG uptake values of the MET group were significantly higher than those of the wdMET group (*P* = 0.049), and those of the wdMET group were significantly higher than those of the noMET group (*P* = 0.003). There were also significant differences (*P* < 0.001) in the average FDG uptake values of the PH, FG, and VMPFC clusters between the three groups. From the MET group to the wdMET group to the noMET group, there was a clear tendency toward higher FDG uptake values (Figure 2(d)). Within the VOIs, the average FDG uptake values of the MET group were significantly lower than those of the wdMET group (*P* = 0.020), and those of the wdMET group were significantly lower than those of the noMET group (*P* = 0.010).

In VOI-based correlation analysis of the imaging findings across the MET and wdMET groups, there was a significant negative correlation (*r* = −0.417, *P* = 0.020) between the individual average FDG uptake values of the three white-matter clusters and metformin withdrawal durations (Figure 2(e)). In contrast, there was a positive but nonsignificant correlation (*r* = 0.280, *P* = 0.127) between the individual average FDG uptake values of the PH, FG, and VMPFC clusters and metformin withdrawal durations (Figure 2(f)). That is, the longer a patient had been withdrawing from metformin, the more reduction of hypermetabolic changes in those affected white matter was. However, in hypometabolic PH, FG, and VMPFC, the association between withdrawal duration and metabolism restoration was not significant.

## 4. Discussion

Previous cellular and animal-model studies prompted the hypothesis that metformin is associated with change in brain metabolism. The previous animal studies [5–8], however, used metformin doses 3–10 times higher than those normally used in humans with diabetes. To explore the effects of a clinical dose of metformin on the brain metabolism in type 2 diabetic patients, we investigated functional changes of FDG PET scan using a voxel-wise analysis.

Unexpectedly, our voxel-wise analysis showed that patients in the MET group had several clusters of significantly increased metabolism in the subgyral white matter of the bilateral cerebral hemispheres, as compared with the patients in the noMET group. A possible explanation for metformin-induced hypermetabolic change of white matter is metformin-related vitamin B12 deficiency, which has been associated with the severity of cerebral white matter lesions [15]. We did not measure our patients' plasma concentrations of vitamin B12 and its related markers beforehand to support the inference of this unexpected finding. However, after we reviewed the complete blood count data (a 2-month window) available from 19 patients in the MET and wdMET groups, we found that none had an elevated mean corpuscular volume or megaloblastic anemia, which is one of the hallmarks of a metformin-induced vitamin B12 deficiency. Consequently, these white matter hypermetabolic changes are less likely caused by metformin-related vitamin B12 deficiency. This hypermetabolic change reasonably might result from metformin-induced inflammation in these white-matter clusters. FDG PET is useful for detecting infection and inflammation [16], including white matter inflammation [17] and therefore can be used to monitor neuroinflammation [18]. Metformin can more than double the production of A*β* [8]. A*β* deposits have been detected in the cerebral white matter of the AD brain, and their distribution corresponded to the orientation of the blood vessels [19]. Activated microglial cells were found colocalized with perivascular deposits of A*β* in the AD brain and seemed to be involved in clearing these deposits [20]. Moreover, hypermetabolism of white matter may be associated with glial cells [21]. High FDG uptake, caused by inflammation as well as microglial cell activation, was detected in immunohistochemical data and indirectly supported by a group of patients with amyotrophic lateral sclerosis with local activated microglia [22, 23]. Therefore, we are of the opinion that A*β*-associated inflammation combined with microglial cell activation causes this hypermetabolic change.

In agreement with our hypothesis, the voxel-wise group analysis found significant hypometabolic regions in the brain memory system caused by a clinical dose of metformin. Compared with the noMET group, the MET group had clusters of significantly decreased metabolism in the PH, FG, and VMPFC, which are part of the semantic memory system [24]. The VMPFC has been linked to motivation, reward processing, learning, and decision making. Lesions of the VMPFC itself affect memory monitoring and induce spontaneous confabulations [25]. Data from monkeys suggest principal connections between the VMPFC (medial orbitofrontal cortex, Brodmann area 11) and the PH areas [26], the other main foci of hypometabolism in the MET group. Patients with such lesions of the posterior medial temporal area were unable to store new information [27]. Characteristically consistent with the results of a meta-analysis [24] of functional neuroimaging studies focusing on semantic processing, the hypometabolic foci in our study were left-hemisphere lateralized. The relatively hypometabolic semantic foci in our study indicated hypofunction of those areas. This finding seems in line with the theory of the potentially harmful consequences of metformin [8, 12] and is supported by a clinical trial [28] which showed that metformin for monotherapy was associated with a declining trend in memory performance.

In the present study, we found that FDG uptake in metformin-influenced foci had significant trends between groups. The regional FDG uptake values used for the association showed a continuous distribution with a broad overlap between the three groups rather than group-centered clusters. This indicates that the association may not have been driven only by group-based differences. Supporting this impression, a significant negative correlation between FDG uptake values of hypermetabolic clusters in white matter and metformin withdrawal durations was also present in the MET and wdMET groups. This suggests that the influence of metformin in white matter is time-dependent on withdrawal duration, that is, on how long it has been since the patient stopped taking metformin. In contrast, there was a weak tendency toward a positive correlation between the FDG uptake values of hypometabolic clusters in semantic foci and metformin withdrawal durations, which indicates that the influence is not time-dependent on the withdrawal duration of metformin or a temporary withdrawal does not reach significant change of these long-lasting effects.

In addition to metformin, two antidiabetic agents, insulin and rosiglitazone, had been investigated for their effect on the link between diabetes and AD [8, 28–30]. In neuronal models, combination usage of insulin reduced the increased intracellular A**β** level caused by using metformin alone [8]. Clinical evidence [30] indicates that intranasal insulin improved cognition in patients with early AD. In clinical trials [28, 29], rosiglitazone ameliorated disease-related pathology and improved memory deficits in animal models of AD; thus, it might protect against cognitive decline in older patients with diabetes. Only one patient in the MET group used insulin and only one patient in the MET group used rosiglitazone as antidiabetic agents. After removing the data of these two patients, we did an additional SPM analysis to verify the first; the results (as shown in the Supplementary Material available online at http://dx.doi.org/10.1155/2014/694326) were similar and consistent with our formal results in Figure 1.

Because this was a pilot imaging study on the effects of metformin on brain metabolism, we were still unable to arrive at exact pharmacological mechanisms that explain some of our findings. One limitation of our study is that we did not measure circulating amyloid levels or apolipoprotein E4 as a dementia-risk allele. Additional studies with more patients and comprehensive examinations such as radionuclide imaging of amyloid, using radiolabeled PK11195 for microglial activation-associated neuroinflammation imaging, and using magnetic resonance imaging for detecting fine white matter abnormalities are needed to arrive at more powerful conclusions. In addition, we focused on metformin's effects in diabetic patients and all the enrolled patients had type 2 diabetes; therefore, we had no data on how metformin affects healthy people. We did not enroll healthy people as a control group because of ethical concerns in administration of metformin to a healthy group. Besides, cerebral glucose metabolism may be influenced to some degree in patients with diabetes. Aiming to explore the effects of metformin in type 2 diabetic patients, it is more reasonable to make comparisons between diabetic patients taking and not taking metformin.

## 5. Conclusions

The clinical dose of metformin in type 2 diabetic patients is associated with hypermetabolic changes in the white matter of the bilateral cerebral hemispheres and with hypometabolic changes in the semantic memory system (the PH, FG, and VMPFC), which is predominantly lateralized in the left hemisphere. Correlation analyses suggested that withdrawal from metformin reduces its effects on white matter hypermetabolic changes, which are duration-dependent. However, restoring hypometabolism in the PH, FG, and VMPFC was not significantly correlated with metformin withdrawal duration.

## Supplementary Material

We did an additional statistical parametric mapping (SPM) analysis to compare cerebral FDG uptake between MET-2-group patients (removing one patient who used insulin and one who used rosiglitazone from the MET group), noMET-group patients (not taking metformin), and wdMET-group patients (withdrawing from metformin). The voxel-wise comparisons included age, BMI, fasting blood sugar, and education as nuisance variables. All results are presented at a threshold of P < 0.005, uncorrected, k > 150 voxels. These metabolic differences between groups (Supplementary Figure) are similar to the results of the first analysis (Figure 1).

## Figures and Tables

**Table 1:** Patient characteristics.

	MET (*n* = 18)	wdMET (*n* = 13)	noMET (*n* = 9)
Gender (M : F)	8 : 10	9 : 4	6 : 3
Age (years)	63.8 ± 7.1	58.7 ± 8.3	60.8 ± 5.6
BMI (kg/m^2^)	25.8 ± 3.4	26.5 ± 2.4	27.7 ± 5.5
HbA_1c_ (%)	7.0 ± 1.0	7.1 ± 1.1	6.3 ± 0.7
Fasting blood sugar (mmol/L)	7.5 ± 1.4	7.3 ± 1.1	7.4 ± 1.4
Metformin daily dose (mg)	1194 ± 546	1058 ± 560	—
Diabetes duration (years)	8.2 ± 7.1	7.9 ± 8.2	7.1 ± 9.0
y < 5 : y ≥ 5	7 : 11	8 : 5	5 : 4
MMSE score	25.6 ± 4.3	27.0 ± 2.2	27.0 ± 2.9
Education (years)	9.7 ± 6.4	9.9 ± 6.2	10.4 ± 5.1
Current smoker	1	2	2

Values are means ± standard deviation or numeric proportions, as indicated. MET: patients taking metformin; wdMET: patients withdrawing from metformin for more than 3 days; noMET: patients not taking metformin; BMI: body mass index; HbA_1c_: glycated hemoglobin; MMSE: Mini-Mental State Examination. There were no significant differences between the three groups (all *P* > 0.05).

**Table 2:** SPM results of the difference in FDG metabolism between groups.

Comparison groups	Cluster size	Voxel level			Anatomic locations
		*T* value	*Z* score	MNI coordinates (*x*, *y*, *z*)	
MET > noMET	421	4.58	3.77	48, −22, −10	Right temporal lobe, subgyral white matter
		3.38	2.99	42, −44, −14	
	401	4.70	3.84	−40, −68, 0	Left occipital lobe, subgyral white matter
		4.51	3.73	−34, −54, −6	Left temporal lobe, subgyral white matter
	683	3.87	3.32	30, 14, 40	Right frontal lobe, subgyral white matter
		3.84	3.31	18, 10, 38	Right limbic lobe, white matter
		3.84	3.30	20, −2, 46	

MET < noMET	217	4.35	3.63	−24, −32, −14	Left limbic lobe, parahippocampal gyrus
	160	4.20	3.54	−48, −44, −26	Left temporal lobe, fusiform gyrus (BA 37)
	623	4.94	3.98	10, 56, −22	Right frontal lobe, orbital gyrus (BA 11)
		4.00	3.41	−8, 34, −26	Left frontal lobe, rectal gyrus (BA 11)
		3.16	2.83	10, 28, −20	Right frontal lobe, medial frontal gyrus (BA 25)

wdMET > noMET	230	4.99	3.82	50, −26, −12	Right temporal lobe, subgyral white matter
	237	3.89	3.21	−40, −62, −4	Left occipital lobe, subgyral white matter
		3.05	3.11	−34, −74, −2	
		3.73	3.11	−38, −54, −2	Left temporal lobe, subgyral white matter
	249	4.22	3.41	20, 2, 46	Right frontal lobe, subgyral white matter
		3.27	2.82	18, −18, 48	

wdMET < noMET	175	5.26	3.95	−30, −34, −22	Left limbic lobe, parahippocampal gyrus

MET > wdMET	340	4.07	3.53	28, 14, −14	Right frontal lobe, subgyral white matter

MET < wdMET	—	—	—	—	No suprathreshold cluster

Threshold at *P* < 0.005, uncorrected, *k* > 150 voxels. MET: patients taking metformin; wdMET: patients withdrawing from metformin for more than 3 days; noMET: patients not taking metformin; BA: Brodmann area.
