# Supplementary material for: Effects of Metformin on the Cerebral Metabolic Changes in Type 2 Diabetic Patients
Source: ScientificWorldJournal. 2014 Mar 24;2014:694326. doi: 10.1155/2014/694326 (PMC3982461; doi:10.1155/2014/694326)
Supplement: Supplementary file 1 — We did an additional statistical parametric mapping (SPM) analysis to compare cerebral FDG uptake between MET-2-group patients (removing one patient who used insulin and one who used rosiglitazone from the MET group), noMET-group patients (not taking metformin), and wdMET-group patients (withdrawing from metformin). The voxel-wise comparisons included age, BMI, fasting blood sugar, and education as nuisance variables. All results are presented at a threshold of P < 0.005, uncorrected, k > 150 voxels. These metabolic differences between groups (Supplementary Figure) are similar to the results of the first analysis (Figure 1). [file 694326.f1.pdf]

Supplementary Material

We did an additional statistical parametric mapping (SPM) analysis to compare cerebral FDG uptake between MET-2-group patients (removing one patient who used insulin and one who used rosiglitazone from the MET group), noMET-group patients (not taking metformin), and wdMET-group patients (withdrawing from metformin). The voxel-wise comparisons included age, BMI, fasting blood sugar, and education as nuisance variables. All results are presented at a threshold of $P < 0.005$, uncorrected, $k > 150$ voxels. These metabolic differences between groups (Supplementary Figure) are similar to the results of the first analysis (Figure 1).

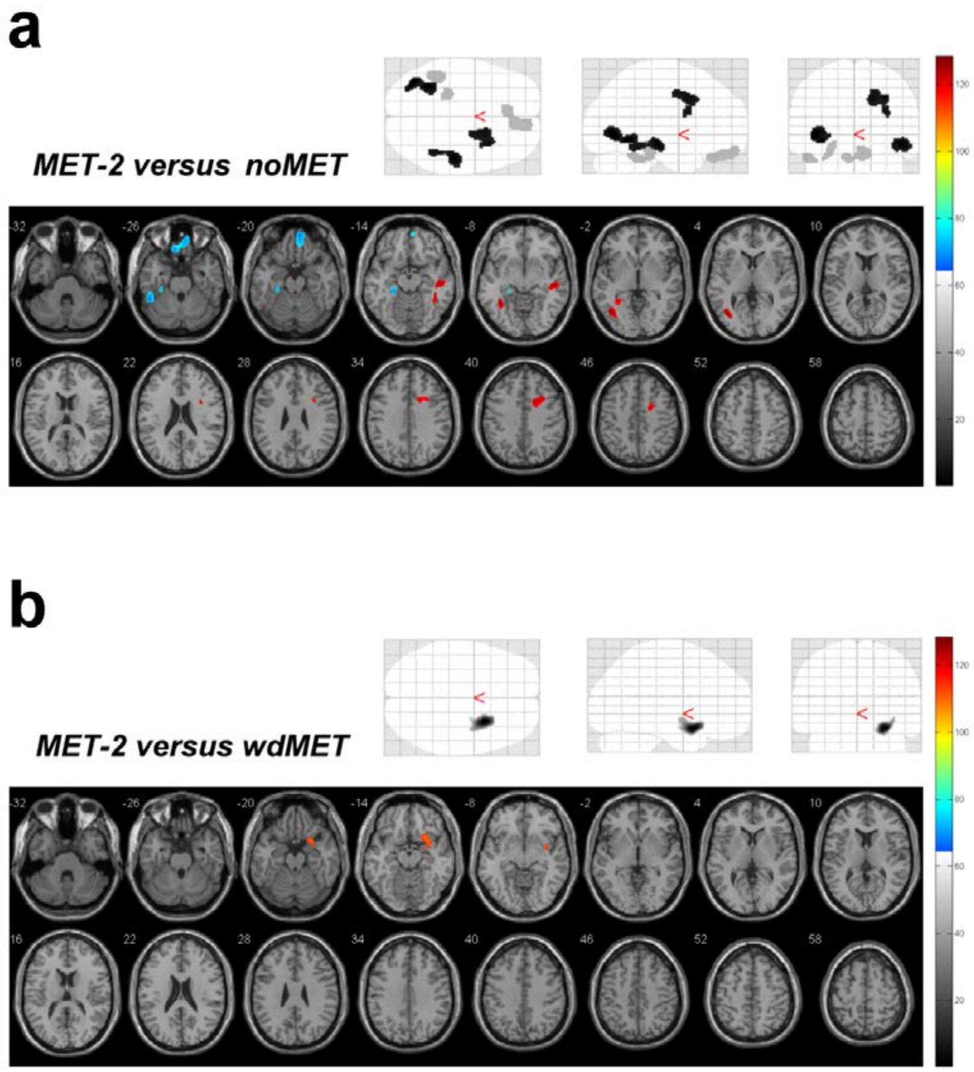

Supplementary Figure (a) MET-2 versus noMET; **(b)** MET-2 versus wdMET. *Top*: SPM glass brain render of the statistical voxel-wise comparisons (black: hypermetabolic regions in the former; light gray: hypometabolic regions in the former). *Bottom*: SPM results, slice renders of

the significant clusters displayed on a T1 template overlaid with magnetic resonance images by SPM5 extension xjView 8.1 (red: hypermetabolic regions in the former; blue: hypometabolic regions in the former).
